# Overprescribing antibiotics for asymptomatic bacteriuria in older adults: a case series review of admissions in two UK hospitals

**DOI:** 10.1186/s13756-019-0519-1

**Published:** 2019-05-02

**Authors:** Emily Rousham, Michael Cooper, Emily Petherick, Paula Saukko, Beryl Oppenheim

**Affiliations:** 1 School of Sport, Exercise and Health Sciences, Loughborough University, Loughborough, Leicestershire, LE11 3TU UK; 2 Department of Microbiology, The Royal Wolverhampton NHS Trust, Wolverhampton, UK; 3 School of Social Sciences, Loughborough University, Loughborough, UK; 4 NIHR Surgical Reconstruction and Microbiology Research Centre, Queen Elizabeth Hospital, Birmingham and Birmingham Community Healthcare Trust, Birmingham, UK

**Keywords:** Urinary tract infection, Diagnosis, Antimicrobial agents, Antibiotic resistance, Guidelines

## Abstract

**Background:**

Overdiagnosis and overtreatment of urinary tract infection (UTI) with antibiotics is a concern. In older adults, diagnosis of UTI using near-patient urine tests (reagent strip tests, dipsticks) is advised against because the age-related increase in asymptomatic bacteriuria can cause false-positive results. Instead, UTI diagnosis should be based on a full clinical assessment. Previous research lacks systematic information on urine dipstick use in hospitals. The aim of this study was to examine the use of urine dipstick tests and microbiology among older adult hospital admissions in relation to recommended UTI diagnostic criteria. A further aim was to assess factors associated with the use of dipsticks.

**Methods:**

A case series review of patients aged ≥70 years admitted to two NHS Trust hospitals in England. Records from 312 patients admitted in 2015 meeting inclusion criteria were selected at random.

**Results:**

Of 298 complete patient records, 54% had at least one urine dipstick test recorded. 13% (21/161) of patients who received a urine dipstick test were diagnosed as having a UTI, only 2 out of these 21 cases had two or more clinical signs and symptoms. 60 patients received a second dipstick test, leading to 13 additional cases of UTI diagnosis. Dipstick tests were more likely to be performed on patients with a history of falls (OR 1.93, 95% CI:1.21, 3.07, *p* < 0.01), and less likely on those with dementia (OR 0.44, 95% CI: 0.22, 0.87, *p* < 0.05). The most common reason for testing was routine admissions policy (49.1% of cases), but these cases were predominantly in one hospital.

**Conclusions:**

Use of urine dipstick tests was high among older adults admitted to hospitals. Most cases were asymptomatic and therefore received inappropriate antibiotic therapy. This paper highlights the need to implement new Public Health England diagnostic guidelines to hospital admission and emergency departments.

## Background

Overdiagnosis and overtreatment can be understood as a healthcare quality problem [1]. In the case of antimicrobial prescribing this is particularly important in order to reduce harm, promote stewardship and avoid inappropriate treatment. Urinary tract infections (UTIs) are the second most common reason for antibiotic prescribing in the UK [2] and diagnosing UTIs in older adults (> 65 years) is recognised as being complex [3, 4]. An estimated 40% of cases of UTI in older people are incorrectly diagnosed [5]. Urine dipsticks (reagent strip tests) are an inexpensive and immediate near-patient test which detect bacteriuria via nitrate and leukocyte esterase in urine. Urine dipsticks and microbiology are both unable to distinguish between asymptomatic bacteriuria (a urine culture with significant growth in an asymptomatic individual), and symptomatic urinary tract infection. Asymptomatic bacteriuria (ASB) increases with age and is sufficiently common in older adults that urine culture ceases to be a reliable diagnostic test [6]. Current guidelines, therefore, recommend the best diagnostic indications for UTIs in adults as the presence of two or more clinical symptoms (dysuria; urgency; frequency, urinary incontinence; shaking chills; flank or suprapubic pain; new onset or worsening of pre-existing confusion /agitations) [7]. Guidelines direct against the use of urine dipsticks and recommend urine microbiology only if signs and symptoms are present to guide prescribing [7]. Similarly, the NICE quality standard (QS260) for older adults recommends that UTIs are diagnosed by a full clinical assessment, without urine dipstick testing, because of varying accuracy [6]. Antibiotic therapy for ASB has been shown to have no clinical benefit [8, 9] and may cause adverse side effects in hospitalised patients such as the development of difficult to treat infections, increased risk of *Clostridium difficile* infection and damage to the microbiome, as well as adding to selective pressure for antimicrobial resistance in healthcare settings [10]. It is increasingly recognised that excess prescribing may be driven by the routine use of reagent strip tests [11]. Previous studies of UTI diagnosis in hospitalised older adults have examined the use of microbiological culture and antibiotic sensitivity testing [12] but have not reported on the use of urine dipsticks. There are significant gaps in knowledge about the routine use of dipsticks and urine microbiology in hospital admissions and the diagnostic pathways for UTIs and antibiotic prescribing. Urine dipstick use is largely undocumented because it is often not captured through routine data.

This study aimed to examine the use of dipsticks and urine microbiology among adults aged ≥70 years admitted to acute and community hospitals using a retrospective case series review. The specific objectives were to understand at a granular level 1) the proportion of older adults who undergo urine reagent strip tests (dipsticks) or urine microbiology on admission 2) the proportion of older adults diagnosed in line with SIGN guidelines and the NICE quality standard (a full clinical assessment followed by microbiological analysis and appropriate treatment) and 3) the proportion of cases with asymptomatic bacteriuria that are treated with antibiotics. A further aim was to identify factors associated with the use of urine dipsticks in order to highlight areas of practice for quality improvement.

## Methods

### Study design

We conducted a retrospective case series review of patient records of adults aged ≥70 years from two NHS hospitals. The study was conducted from January to August 2017 after obtaining Health Research Authority approval to extract data (IRAS approval reference 202255). Data were extracted from patient admissions to eventual diagnosis/discharge pertaining to the diagnosis of urinary tract infections.

The primary outcome was the proportion of older adults (≥70 years) who were diagnosed with UTI and prescribed antibiotics as a result of dipstick tests and the proportion diagnosed through urine microbiology in comparison to recommended diagnostic criteria (two or more signs or symptoms of UTI with confirmatory microbiology).

Two patient and public involvement (PPI) groups (Leicester Academy for the Study of Ageing and Queen Elizabeth Hospitals PPI) were consulted on the study design, data collection methods, activities to raise public awareness of the study and disseminate findings, before applying to the Health Research Authority (HRA). During the consultation period an age-group of > 70 years was recommended for this study based on feedback from clinicians in the two hospitals. The potential benefits of the research to patient care were considered to be justified in a frail older group. Study findings were disseminated face-to-face at PPI meetings, in writing to the NHS Trusts and PPI groups, and on the website of the NHS Trust Sponsor.

### Study setting and participants

The study was carried out in one acute care and one community hospital in England. Birmingham Community Healthcare NHS Foundation Trust (BCHC) is an intermediate care facility while New Cross Hospital of The Royal Wolverhampton NHS Trust (RWT) is a large general hospital with approximately 800 beds.

Inclusion criteria were patients aged ≥70 years admitted to either hospital from 1 January 2015 to 30 June 2015. Where a patient had more than one hospital admission during the study period only the first admission was included.

### Sample size

The target sample size was 250 patient records; 125 records from each site. The sample size calculation was based on existing admissions data. In 2015, New Cross Hospital had approximately 3800 admissions per month for patients aged ≥70 years. BCHC had a total of 4227 admissions of adults aged ≥70 years in 2014/15 with a UTI prevalence of 18% in the hospital population. With an estimated population size of 800 and a 20% prevalence of UTI (95% confidence level, 5% error) a sample size of 119 patient records at each site would be adequate for statistical analyses. Our target sample size therefore was rounded to 250 patient records.

### Sampling technique

A research nurse liaised with NHS Information Services to extract the IDs of patient records meeting inclusion criteria at each site. From this list, the required number of case records was selected using the random number generator function in Excel and corresponding medical records were requested.

### Data extraction

Data were extracted on date of admission, age at admission, existing co-morbidities, history of falls and clinical signs and symptoms of UTI [6]. Data on collection of urine samples, urine dipstick tests performed and results recorded, urine microbiological results including culture, organism identified, susceptibility and antibiotics prescribed were recorded. No personal identifying information was extracted.

In all cases, we use the term ‘diagnosed with UTI’ based on exactly what was recorded in patient notes. The presence or absence of a UTI based on the patient record was extracted along with all dipstick test results, all microbiology results, and all signs and symptoms recorded. The investigators did not make any independent assessment or diagnosis.

### Statistical analysis

Data were analysed using IBM SPSS Version 23.0. Patients receiving urine dipsticks and microbiology were expressed as proportions and percentages out of the total number of cases. Unadjusted odds ratios and 95% confidence intervals were calculated to identify predictors of receiving a urine dipstick test in univariate analyses. The positive and negative predictive values of dipstick tests compared to microbiology were calculated.

## Results

**Fig. 1:**
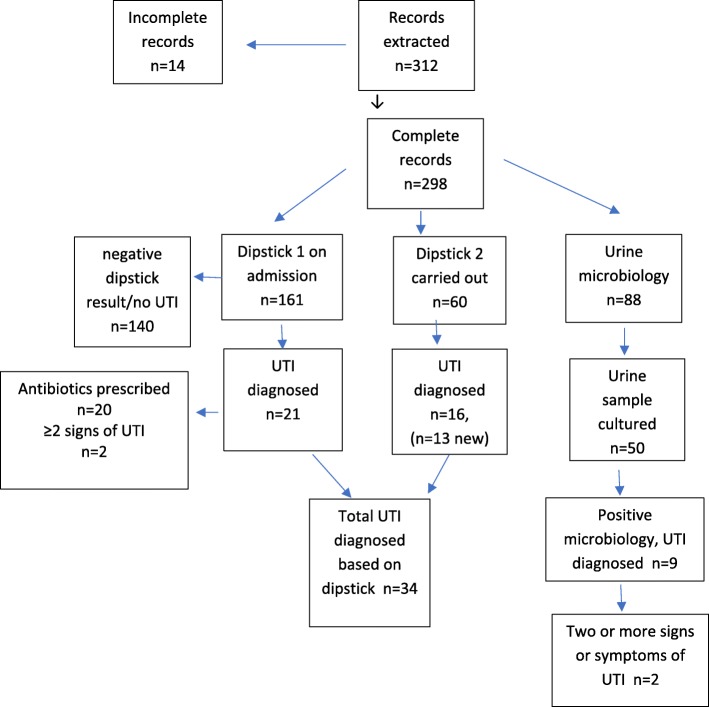
Summary of urine dipstick tests, microbiology and reported UTI from records (*n* = 312)

A total of 312 patient records were reviewed, of which 14 records (4.5%) had missing medical records leaving 298 available for analysis (Fig. 1). The mean age of the sample was 83.6 (SD 7.25) years, with 70.5% (*n* = 210) female patients. The most prevalent comorbidities in the sample were cardiovascular disease (69.5%, *n* = 207), musculoskeletal disease (36.2%, *n* = 108), cerebrovascular disease (25.2%, *n* = 75) and diabetes (25.1%, *n* = 41) (Table 1).

**Table 1:** Sample demographic characteristics, co-morbidity and urinary tract infection signs and symptoms

	Percent	(Number)
Age in years, mean (SD)	83.6 (7.25)	(298)
Gender, % female	70.5	(210)
Leading co-morbidities		
Cardiovascular disease	69.5	(207)
Musculoskeletal disease	36.2	(108)
Diabetes	25.1	(79)
Cerebrovascular disease	25.2	(75)
Dementia	13.8	(41)
Living independently at time of admission	83.0	(247)
Recent history of falls	50.3	(149)
Fully continent	55.7	(166)
Indications for urine dipstick test (more than one response in some cases (*n* = 171)		
Confusion	2.9	(5)
Clinical state	22.8	(39)
Fall	3.5	(6)
Smell/odour	0.6	(1)
Routine admission policy	49.1	(84)
Reason not recorded	21.1	(36)
UTI signs and symptoms recorded		
Dysuria	1.0	(3)
Frequency	4.7	(14)
Incontinence	4.7	(14)
Shaking/chills	0.7	(2)
Flank or suprapubic pain	1.0	(3)
Frank haematuria	0.3	(1)
New/worsening of existing confusion or agitation	3.7	(11)
Other^a^	1.0	(3)
Total signs and symptoms of UTI		
None	84.5	(253)
One	13.7	(41)
Two or more	1.3	(4)

^a^Three cases of ‘Other’ were: lower back pain, nephrostomy and reduced urine output

On admission to hospital, 54% of patients (161/298) had a dipstick test recorded, of whom 13.0% (21/161) were diagnosed according to the dipstick result as having UTI and 12.4% (20/161) of these were prescribed antibiotics based on the positive dipstick result. The antibiotics prescribed were: coamoxiclav (*n* = 3); trimethoprim (*n* = 15); nitrofurantoin (*n* = 1) and pipercillin/tazobactam (*n* = 1). This did not include any of the patients who had been diagnosed with a UTI prior to admission (*n* = 21). The most common indications for conducting a dipstick test as written in patient notes were ‘routine practice’ (49.1%) followed by ‘clinical state’ 22.8% and ‘other’ 21.1% (Table 1). When data were disaggregated by hospital, almost all cases of dipstick use as routine practice were at BCHC (95.2%, 80/84). 23 out of 161 patients were catheterised at the time of the urine dipstick test being carried out. One of these (1/23) was diagnosed with a UTI based on the dipstick test result.

The most common signs and symptoms of UTI recorded were ‘frequency’ and ‘incontinence’ (Table 1). Of the total sample, 1.3% (*n* = 4) had two or more signs or symptoms of UTI; 13.7% (*n* = 41) of patients had one sign or symptom of UTI, and 84.5% (*n* = 253) had no signs or symptoms. Of those diagnosed as having UTI based on a dipstick test (*n* = 21), 11 cases (52.4%) had no symptoms, eight cases (33.1%) had one symptom and two cases (9.5%) had two or more symptoms.

Sixty patients (20.1% of total sample) had a second dipstick test recorded, 26.7% (16/60) of whom were diagnosed as having UTI based on the dipstick test. Thirteen of these were new cases that had not been diagnosed in the first dipstick result. Those diagnosed with a UTI on the second dipstick test results were prescribed: coamoxiclav (*n* = 1); trimethoprim (*n* = 13) and ciprofloxacin (*n* = 1).

Altogether, 34 patients were diagnosed as having a UTI based on 220 known dipstick tests, representing 15.4% of the sample.

### Predictors of urine dipsticks and UTI diagnosis by dipstick

Patients were significantly more likely to undergo a urine dipstick test if they had a history of falls (62.4% vs 46.3%, OR 1.93; 95% CI 1.21, 3.07) and significantly less likely to have a dipstick test if they had dementia (36.6% vs 56.8%, OR 0.44; 95% CI 0.22, 0.87). Patients admitted to RWT were significantly less likely to have a dipstick test compared with those admitted to BCHC (35.7% vs 68.0%, OR 0.26; 95% CI 0.16, 0.42) (Table 2), reflecting the reasons given in notes of giving dipstick tests as part of routine care. There was no difference in the proportion of patients receiving dipsticks among males and females, among those living independently compared to those in nursing homes or residential care prior to admission, and those with musculoskeletal disease or diabetes.

**Table 2:** Univariate analysis of predictors of urine dipstick testing on admission to hospital

		Total n	Proportion receiving dipstick test % (n)	OR (95% CI)
Sex	Male	83	47.0 (39)	0.68 (0.41, 1.12)
	Female	215	56.7 (112)	
NHS hospital	RWT	129	35.7 (46)	0.26 (0.16, 0.42)
	BCHC	169	68.0 (115) ***	
Recent history of a fall	Yes	147	62.4 (93)	1.93 (1.21, 3.07)
	No	149	46.3 (68)**	
Living independently	Yes	247	53.4 (113)	0.95 (0.52, 1.76)
	No	51	54.9 (28)	
Musculoskeletal disease	Yes	108	60.2 (65)	1.48 (0.92, 2.39)
	No	190	50.5 (96)	
Dementia	Yes	41	36.6 (15) *	0.44 (0.22, 0.87)
	No	257	56.8 (146)	
Diabetes	Yes	79	49.4 (122)	0.77 (0.46, 1.29)
	No	219	55.7 (39)	

*p < 0.05; **p < 0.01, ***p < 0.001

Comparisons of cases with a positive versus negative dipstick result (and consequently diagnosed as positive or negative for UTI) showed no difference by sex, history of falls, independent living, dementia, musculoskeletal disorders or diabetes (Table 3). Taking a positive dipstick as an indication of ASB, the presence of bacteriuria, therefore, had no association with any of the variables tested.

**Table 3:** Likelihood of urinary tract infection diagnosis among patients receiving a dipstick test

		n	Percent diagnosed as having UTI as a proportion of all those receiving a first dipstick test total *n* = 161	OR (95% CI)
Sex	Male	39	10.3 (4)	0.71 (0.22, 2.24)
	Female	122	13.9 (17)	
Recent history of a fall	Yes	93	13.2 (9)	0.97 (0.38, 2.45)
	No	68	12.9 (12)	
Living independently	Yes	133	12.8 (17)	0.87 (0.27, 2.85)
	No	28	14.2 (4)	
Musculoskeletal disease	Yes	65	12.3 (8)	0.89 (0.35, 2.30)
	No	96	13.5 (13)	
Dementia	Yes	15	13.3 (2)	1.03 (0.22, 4.92)
	No	146	13.0 (19)	
Diabetes	Yes	39	17.9 (7)	1.69 (0.63, 4.54)
	No	122	11.5 (14)	

### Urine microbiology

29.5% of patients (88/298) had notes on microbiological analysis on the first urine sample taken on admission. Of these, 16.7% (50/298) had a sample cultured. Of the total samples cultured, 28% had no growth (*n* = 14), 38% had mixed growth (*n* = 19) and 34% had an organism identified (*n* = 17). Nine cases were diagnosed with a UTI based on urine microbiology, of which 2 cases had 2 or more signs and symptoms of UTI.

The level of agreement between UTI diagnosed by dipstick and microbiology is shown in Table 4. A positive dipstick results was a poor predictor of bacteriuria based on urine microbiology (2/21 = 9% positive predictive value). A negative dipstick result, however, was a stronger predictor of negative urine microbiology, with a 95% negative predictive value (7/140). There were only two cases with a concordant diagnosis of UTI based on both the dipstick test result and the microbiology result from a total of 161 patients receiving a dipstick test.

**Table 4:** Level of agreement between urine dipstick test result and urine microbiology result

UTI diagnosis	Urine microbiology		
Dipstick	UTI^a^ Positive	UTI^a^ Negative	Total
UTI Positive	2	19	21
UTI Negative	7	133	140
Total	9	152	161

^a^UTI positive as noted in patient record, not assigned by investigators

## Discussion

This study has demonstrated widespread use of urine dipstick tests, used on 54% of patients ≥70 years in two NHS hospitals.

13% (21/161) of patients who were tested with a urinary dipstick were diagnosed as having a UTI and 20/21 of these patients were prescribed an antibiotic. Only two of these cases had two or more clinical signs or symptoms of UTI. After including patients who received a second dipstick test, 15.4% (34/220) of the total sample were diagnosed as having a UTI based on a dipstick alone. This detailed analysis indicates that the majority of those diagnosed and treated with antibiotics had ASB rather than UTI and were therefore given inappropriate therapy. Given that hospital admission episodes for the 65–84 age group has seen the greatest increase in England over the last ten years, with 6.3 million total episodes in 2016–17 [13], these practices are likely to be a significant contributor to overdiagnosis and treatment in England.

Other factors associated with the use of dipsticks such as history of falls are more likely associated with healthcare cultures of ‘prevention better than cure’ [1]. Ease of obtaining a urine sample may be the underlying reason for dementia patients being less likely to receive a dipstick rather than any clinical features. Half of all dipstick use (49.1%) was driven by routine admissions policy, but almost all these cases were from one hospital demonstrating significant variation in diagnostic practices between hospitals.

Urine microbiology appeared to be a lesser contributor to overdiagnosis and overtreatment, nonetheless of the 9 cases (out of 88 with known microbiology) diagnosed as having UTI based on presence of bacteriuria, only 2 of these cases had clinical signs and symptoms of UTI. Studies of nursing homes in the US have also concluded that urine culture results play a significant role in antibiotic overprescribing among older adults [14]. In a US study of hospital admissions of adults of all ages, positive urinalysis and positive urine culture were associated with antibiotic prescribing, but the presence of UTI symptoms or signs was not associated with antibiotics [15].

There are some potential limitations in the study: firstly, the results from two hospitals will not be generalisable to all hospitals in England. Indeed, the marked difference in routine use of dipsticks between the two hospitals highlight the variability in diagnostic practices in NHS Trusts across the country. Secondly, the findings are influenced by documentation in the medical records, for which the accuracy and completeness of information is unknown. However, this is the only systematically recorded source of information on dipstick tests, urine microbiology and signs and symptoms of UTI available. A final important consideration is that practices in hospital admissions may have changed since the time of data extraction and there may have been new initiatives around antimicrobial stewardship or UTI diagnosis.

Strengths of the study include data extraction at a granular level to understand the use of dipsticks, a largely unreported aspect of hospital admissions, and meeting the required sample size to achieve statistical power. The inclusion of a representative sample within two NHS hospitals of all patients aged ≥70 years (rather than examining only diagnosed cases of UTI through routine data) is a further strength. The data illustrate how urine dipsticks and microbiology in older adults lead to the detection of ASB which, in turn, prompts prescription of antibiotic therapy. We present quantitative data on UTI diagnostic processes that provide important complementary information to existing qualitative studies. Such studies have reported that clinical staff in hospitals acknowledge relying on dipsticks and urine culture to test for infection rather than using signs and symptoms [16]. In a survey of physicians, 46% admitted prescribing antibiotics for ASB despite knowing that they were not indicated [16]. The difficulties of using clinical examination to diagnose UTI when patients are frail and unwell or unable to report signs and symptoms is also a significant challenge for healthcare professionals. The potential harm to patients by prescribing antibiotics for ASB, however, is under-recognised [8, 11].

Improvement of current practices is not without challenges. A study of UTI diagnosis among hospitalised older adults reported 31.3% of all UTI cases were diagnosed according to SIGN guidance [12]. After an intervention consisting of feedback to ward managers, changes to proformas and UTI guideline flowcharts displayed on wards, the proportion of appropriately diagnosed UTIs increased to 42.1% but this was not statistically significant [12]. Even after a hospital wide intervention, therefore, the majority of cases were not diagnosed in line with recommendations. However, quality improvement initiatives to reduce or ban the use of urine dipsticks in older adults are gaining momentum, particularly in nursing homes. This promises potential for hospital-based interventions.

## Conclusions

This in-depth case-series review sheds important light on practices and behaviours in two hospitals in England. Recent diagnostic guidelines for UTI by Public Health England clearly advise against the use of dipsticks for men and women over 65 years of age [17]. These guidelines require implementation in hospital admission and emergency departments with ongoing measurement and evaluation of effectiveness. Stopping inappropriate or ineffective practices can be more challenging than implementing new ones, particularly for low-cost tests [1, 18]. The reasons why healthcare professionals continue with practices for which there is little or no evidence include individual beliefs, professional cultures and wider contextual influences [18]. Recommended methods to reduce overdiagnosis and overtreatment in quality improvement research [1] should be applied to UTI diagnosis in older adults. Such approaches include making healthcare patient-centred and responsive to an individual patient’s needs.

## Abbreviations

ASB: Asymptomatic bacteriuria
BCHC: Birmingham Community Healthcare NHS Foundation Trust
PPI: Patient and Public Involvement
RWT: The Royal Wolverhampton NHS Trust
UTI: Urinary tract infection
